# Profil étiologique des anémies dans un service de médecine interne

**DOI:** 10.11604/pamj.2017.26.10.11368

**Published:** 2017-01-04

**Authors:** Ali Zinebi, Hicham Eddou, Karim Mohamed Moudden, Mohamed Elbaaj

**Affiliations:** 1 Service de Médecine Interne, Hôpital Militaire Moulay Ismail, Meknès, Maroc; 2 Service d’Hématologie Clinique, Hôpital Militaire Moulay Ismail, Meknès, Maroc

**Keywords:** Anémie, profil étiologique, anémie ferriprive, médecine interne, Anemia, etiologic profile, iron deficiency anemia, internal medicine

## Abstract

L'anémie constitue un problème majeur de la santé publique à travers le monde malgré l'amélioration remarquable des conditions de vie. Elle est classée par l'OMS comme l'un des dix problèmes les plus sérieux du monde. Notre objectif est de décrire les profils épidémiologique et étiologique des cas d'anémies prises en charge dans notre formation. Il s'agit d'une étude rétrospective menée durant 5 ans, allant de Janvier 2011 à décembre 2015 et portant sur 150 patients. L'âge moyen de nos patients est de 48,8ans et les femmes sont les plus touchées avec un sex-ratio de 1,78. Le taux moyen de l'hémoglobine est de 8 g/dl avec des extrêmes allant de 3,4 à 11,4 g/dl. L'anémie ferriprive est le diagnostic étiologique dominant étant retrouvée dans 60% des cas, suivie par l'anémie mégaloblastique observée chez 21% des patients puis les anémies hémolytiques dans 7,33% des cas. La survenue d'une anémie chez l'adulte peut représenter un véritable défit diagnostique pour l'interniste et cela parfois dans un contexte d'urgence. Le recours à des examens spécialisés peut s'imposer.

## Abstract (en)

Anemia is a major public health problem worldwide despite remarkable improvement in living conditions. The World Health Organization (WHO) classifies it as one of the ten most serious problems in the world. This study aims to describe the epidemiologic and etiologic profiles of the cases of anemia treated during our training. We conducted a retrospective study involving 150 patients carried out over 5 years, from January 2011 to December 2015. The average age of our patients was 48.8 years and women were more likely to be affected than men, with a sex ratio of 1.78. The mean hemoglobin was 8 g/dl, ranging from 3.4 to 11.4 g/dl. Iron deficiency anemia was the leading etiologic diagnosis made in 60% of cases, followed by megaloblastic anemia in 21% of patients and haemolytic anemia in 7.33% of cases. The occurrence of anemia in adults may represent a real diagnostic challenge for the internist as it is sometimes associated with an emergency context. Specialized examinations may be necessary.

## Introduction

Malgré l'amélioration remarquable des conditions de vie durant ces dernières décennies, l'anémie demeure un problème majeur de santé publique en affectant la croissance physique, le développement cognitif, la reproduction et la capacité de travail physique ce qui aboutit à une diminution de la performance humaine. Elle a été classée par l'Organisation Mondiale de la Santé (OMS) comme l'un des dix problèmes les plus sérieux du monde moderne et constitue la forme de carence en micronutriments la plus répandue dans le monde. On estime que, pour l'ensemble du monde, elle atteint le chiffre de 2 milliards d'individus avec une prévalence de 24.8% [1] dont 9 sur 10 vivants dans les pays en voie de développement [2]. Les plus exposés sont les nourrissons, les enfants en période de croissance intensive, les sujets âgés et les femmes enceintes. Dans la plupart des pays en développement où elle serait responsable de la moitié des cas d'anémie, le régime alimentaire courant dans la majorité des ménages ne fournit qu'une biodisponibilité en fer alimentaire de 15-25µg Fe/kg/j. En Afrique et en Asie, l'anémie serait responsable de 3.7% à 12.8% des décès maternels au cours de la grossesse et de l'accouchement [3]. L'anémie est définie par une baisse du taux d'hémoglobine. Les seuils inférieurs d'hémoglobine varient en fonction de l'âge, du sexe d'une personne, de son ethnie, de l'altitude à laquelle elle vit, de ses habitudes tabagiques et du stade de la grossesse [4]. Ils peuvent aussi varier selon les auteurs et selon les laboratoires. L'OMS a défini l'anémie selon des normes quasiment identiques avec des variations modérées pour les moins de 15 ans, comme le montrent le Tableau 1 [5]. La découverte d'une anémie doit conduire à un bilan étiologique précis et orienté par les données cliniques et biologiques. L'anémie ou plutôt les anémies, sont le fait de causes et mécanismes physiopathologiques variés et complexes qui rendent certains diagnostics intriqués et difficiles. En effet, à 1 an, seulement 30% des patients anémiques ont un diagnostic étiologique. Les carences en fer, folates et vitamines B12 représentent les causes principales de l'apparition de l'anémie. La présente étude vise à établir un profil épidémiologique, clinique, biologique et étiologique des anémies dans un milieu hospitalier marocain, notamment au service de médecine interne de l'Hôpital Militaire Moulay Ismail Meknès au Maroc.

**Tableau 1:** Définition de l’anémie par l’OMS selon l’âge et le sexe

Age et sexe	Seuil d’hémoglobine (g/dL)
Enfant 6 à 5 ans	11
Enfant 5 à 11 ans	11,5
Enfant 12 à 14 ans	12
Femmes non enceintes (> 15 ans)	12
Femmes enceintes	11
Hommes (> 15 ans)	13

## Méthodes

Il s'agit d'une étude rétrospective s'étalant sur une période de cinq ans allant de janvier 2011 à décembre 2015. Les observations analysées sont colligées au niveau service de médecine interne à l'hôpital militaire Moulay Ismail de Meknès. On a inclut les patients ayant consulté pour un syndrome anémiques avec comme seuil un taux d'Hémoglobine inférieur à 13g /dl chez les hommes et inférieur à 12g/dl chez les femmes. 150 observations d'anémie ont été ainsi colligées. Nous avons exclu de cette étude les anémies de découverte fortuite ou survenue chez des patients avec une étiologie connue.

## Résultats

**Figure 1:**
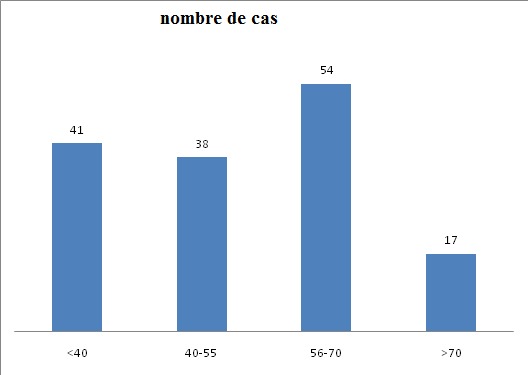
Répartition des patients selon les tranches d’âges

**Figure 2:**
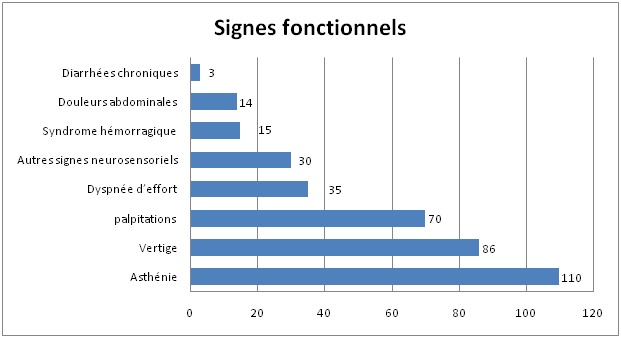
Caractéristiques des patients selon leurs signes fonctionnels

**Figure 3:**
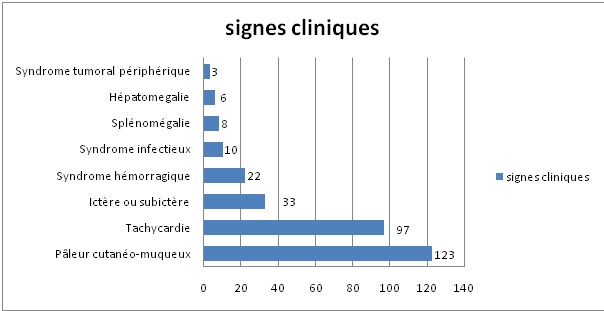
Caractéristiques des patients selon leurs signes cliniques

**Figure 4:**
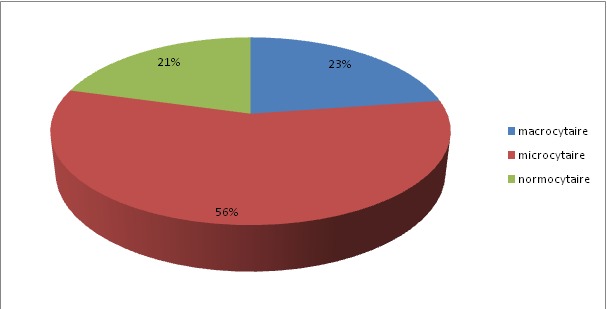
Répartition des anémies en fonction du VGM

**Figure 5:**
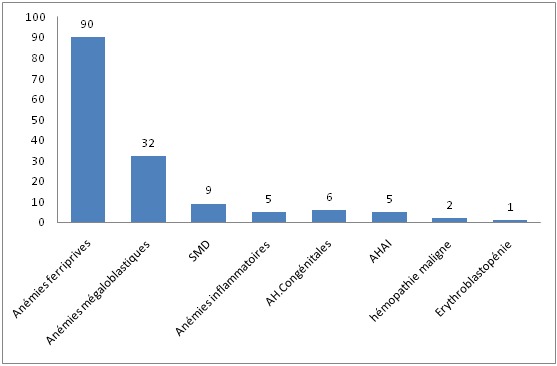
Répartition des cas selon l’origine des anémies

**Figure 6:**
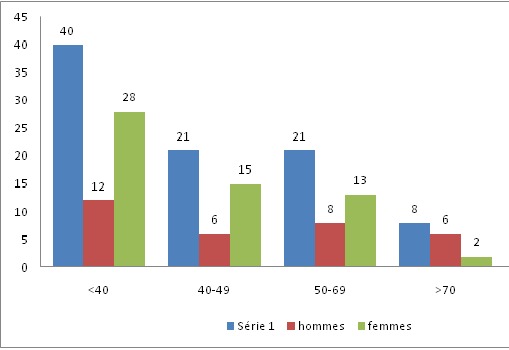
Répartition des anémies ferriprives selon les tranches d’âge et du sexe

Durant la période d´étude, 150 cas d'anémies ont été recensés. L'âge moyen est de 48,4ans avec des extrêmes allant de 14 ans à 80 ans. La Figure 1 montre la répartition des patients en fonction de la tranche d'âge. Les femmes sont les plus touchées avec un sexe ratio F/H de 1,78. Le délai moyen de consultation était de 5mois avec des extrêmes allant de 4 semaines à 2ans. L'asthénie est le premier motif de consultation retrouvée chez 41% des patients de notre série, suivie par la pâleur (Tableau 2). Les différents signes fonctionnels ainsi que physiques rapportés par les patients au moment du diagnostic sont résumés dans les Figure 2 et Figure 3. Sur le plan biologique, le taux moyen de l'Hb est de 8 g/dl, avec des extrêmes allant de 3.4 g/dl à 11,4 g/dl. Une anémie sévère avec un taux d'Hb de moins de 6g/dl a été trouvée chez 20% des cas. L'étude des autres paramètres de l'hémogramme nous a permis de distinguer les différents types d'anémies. L'anémie hypochrome microcytaire vient largement en tête (56% des anémies trouvées) (Figure 4 ). Le taux de réticulocytes supérieur à 120Giga/l a été retrouvé chez 9 patients (soit 6% des cas). A côté de l'anémie, nous avons relevés d'autres anomalies hématologiques chez d'autres patients de notre série à savoir : thrombopénie chez 9 patients, thrombocytose chez 10 patients, neutropénie chez 6 patients et leucocytose chez 5 patients. Le myélogramme a été réalisé chez les patients présentant une anémie normo ou macrocytaire arégénérative après avoir éliminé une carence en vit B12 ou martiale. Il a permis de faire le diagnostic de : 9 cas de syndrome myélodysplasique (SMD), 2 cas de d'excès plasmocytaire (> 10%) et 1 cas d'érythroblastopénie. Un bilan martial à base de dosage de la ferritinémie a été demandé chez les 85 patients ayant une anémie hypochrome microcytaire mais aussi chez certains patients normochrome normocytaire arégénérative. Chez dix patients, on a eu recours au dosage du récepteur soluble de la transferrine. Ce bilan a été indiqué chez les patients ayant une anémie hypochrome microcytaire avec un taux de ferritine normale ou élevé et chez certains patients avec anémie normochrome normocytaire arégénérative. Ce bilan nous a permis de confirmer l'origine inflammatoire de l'anémie chez 5 patients. Un dosage vitaminique (dosage Vit B12 et Vit B9) a été fait chez les patients ayant une anémie macrocytaire arégénérative. Ce dosage a permis de faire le diagnostic d'une carence vitaminique chez 32 patients répartis comme suit : 31 patients ont des taux de vitamine B12 inférieurs à 200 µg/L. Un déficit en folates a été objectivé chez une patiente (moins de 5 ug/L). Un bilan d'hémolyse (bilirubine, LDH et haptoglobine) a été réalisé chez les patients avec anémie normochrome normocytaires régénératives et chez les patients avec anémie microcytaire ayant un taux de ferritine normal sans syndrome inflammatoire. Des stigmates d'hémolyse ont été retrouvés chez 11 patients. En se basant sur les données cliniques et l'apport des différents examens paracliniques, de nombreuses origines pour ces anémies ont été identifiées dans notre étude comme on peut le constater sur la Figure 5. Anémie par carence martiale est le type d'anémie le plus fréquent dans notre étude, retrouvé chez 90 patients (soit 60% des anémies recensées). Les femmes sont les plus touchées avec un sexe ratio F/H de 2. L'âge moyen dans ce groupe est de 40,5 ans. On constate que ce genre d'anémie intéresse les femmes jeunes et les sujets âgés de sexe masculin (Figure 6 ). Les anémies mégaloblastiques constituent la deuxième cause d'anémie dans notre étude, et on a recensé 32 cas (soit 21,33%) de l'ensemble des étiologies. La moyenne d'âge dans ce groupe est de 50,47 ans avec des extrêmes allant de 22 ans à 73 ans. Les femmes sont les plus touchées (21 femmes soit 72% des cas), avec un sex-ratio F/H de 2. L'anémie hémolytique constitue la troisième cause d'anémie dans notre série. On a recensé 11 cas dont la majorité faite de femmes (8). L'âge variait entre 18ans et 58ans avec une moyenne d'âge de 32 ans. Le bilan a permis de classer ces anémies en une anémie hémolytique auto-immune chez 5 patients : 3 cas d'A.H.A.I. idiopathique, 1 cas A.H.A.I. secondaire à un lupus, un cas de syndrome d'Evans chez une patiente ayant une thrombopénie associée. Une hémoglobinopathie a été notée chez 4 patients avec 2cas d'hémoglobinose C, une drépanocytose cosmopolite chez une patiente, un cas de béta-thalassémie intermédiaire et un déficit en G6PD chez deux personnes. Quatrième étiologie par ordre de fréquence est représentée par Syndrome myélodysplasique, puisque recensée chez 9 patients dont la majorité était des hommes (8 hommes). Leur moyenne d'âge était de 69 ans. Un seul patient avait des antécédents de cancer du poumon traité par antimitotiques 10 ans auparavant. Les données de l'hémogramme, du myélogramme et du caryotype ont permis de les classer en : 5 cas de cytopénie réfractaire avec dysplasie multilignée, 1 cas d'anémie réfractaire avec sidéroblaste en couronne, 1 cas de d'anémie réfractaire avec excès de blaste et des anomalies complexe au caryotype, 1 cas de Leucémie myélo-monocytaire chronique et 1 cas de dyserythropoiese congénitale lié à l'X chez un enfant de 8 ans. Cinquième cause des anémies est l'anémie inflammatoire notée dans notre série chez 5 patients dont 3 femmes et deux hommes. L'âge variait entre 20ans et 70 ans avec une moyenne d'âge de 41,26ans. Un Bilan à visée étiologique a été réalisé chez ces patients retrouvant 3 cas de tuberculose, 1 cas de lymphome non hodgkinien et 1 cas de tumeur digestive. Une érythroblastopénie a été retenu chez un seul patient âgé de 45ans. Aucune étiologie n'a été retrouvée malgré un bilan étiologique exhaustif.

**Tableau 2:** Répartition des patients selon le motif de consultation

Motif d’hospitalisation	Nombre	Pourcentage (%)
Asthénie	61	41
Fortuite	30	20
Pâleur	23	16
Dyspnée d’effort	19	12
Altération de l’état general	11	7,3
Ictère cutanéomuqueux	6	4

## Discussion

Dans cette partie nous allons discuter surtout l'anémie ferriprive, le type d'anémie le plus fréquemment rencontré dans notre série et dans la littérature. La recherche étiologique constitue une étape surtout longue et aussi parfois difficile de la prise en charge La prévalence de l'anémie en 2010 dans la population mondiale reste de 33% avec deux pics d'âge : avant 5 ans et après 65 ans [6]. L'anémie ferriprive (AF) est définie comme tout état pathologique dans lequel la teneur en sang de l'hémoglobine est devenue anormalement faible, à la suite d'une carence en fer. L'AF est le type d'anémie le plus répandu au monde, ce qui confirme nos résultats et comme l'a montré Kassebaum NJ [6]. Ce qui rejoint nos résultats puisque l'AF vient en tête des types d'anémies dans notre série avec plus de la moitié des cas recensés. Pour Ben Ahmed, dans une étude dans la région du Cap Bon tunisien portant sur 40 cas [7], la carence martiale est prédominante dans 60% des cas et est liée le plus souvent à une alimentation carencée dans 83%, une cause gynécologique (8,4%) ou un saignement digestif est notée dans 8,4% des cas. Le déficit martial est le déficit nutritionnel le plus répandu au niveau mondial et atteindrait 1 milliard d'individus. Il concerne à la fois les pays en voie de développement, et aussi les pays industrialisés, au point que certains d'entre eux ont mis en place des programmes de prévention avec supplémentation des groupes à risque et enrichissement en fer de certains aliments. La prévalence de la carence en fer dépend de : L'âge : avec un risque plus élevé pour les enfants prématurés, en période de croissance puisque 43% des jeunes enfants dans le monde sont touchés (87% dans les pays en développement contre 26% aux pays industrialisés) [8] et chez les sujets âgés (18 cas recensés dans notre étude soit 20% des AF). Dans notre série l'âge moyen était de 40,5 ans et Ruivard [9], dans une étude portant sur 299 patients suivis en Médecine interne, a retrouvé un âge moyen de 68+/-14 ans. Dans une étude tunisienne menée dans un service de médecine interne [10], l'âge moyen était de 48.81+/-19.85 ans. Selon McLean E [1], l'anémie touchait plus de 47% des enfants de moins de 5 ans au niveau mondial. Ce taux est d'environ 40% en Amérique du Sud, 17% en Europe et atteint 64,6% sur le continent africain, ce qui représente plus de 90 millions d'enfants. Cette carence altère donc la capacité d'apprentissage des enfants et par conséquent leur insertion sociale et économique ultérieure de même que leur croissance et les défenses immunitaires [11]. L'éradication de la carence en fer constitue, pour ces raisons une priorité de santé publique et une des grandes causes sanitaires internationales [8]. L'ignorance des facteurs étiologiques surtout dans les pays en voie de développement limite ainsi la portée des stratégies mises en œuvre [12].

Du sexe avec une prédominance chez les femmes surtout en activité génitale lié à la grossesse, l'allaitement et aux menstruations [8], en raison d'une augmentation des besoins en fer. Dans notre série une prédominance féminine a été établie, ce qui concorde avec les données de la littérature. La prédominance féminine a été notée dans 62,37% selon Ruivard [12]. Dans une étude nord-américaine [13], 11% des hommes de plus de 65 ans et 10,2% des femmes de cette tranche d'âge sont anémiques. Dans d'autres études [14], la prévalence de la carence martiale atteint 60% des hommes et 40% des femmes au-delà de 85 ans. Ceci pourrait s'expliquer par institutionnalisation, faible niveau socio-économique, présence de nombreuses comorbidités [15]. La prévalence globale de l'anémie ne dépend pas du sexe de l'enfant, mais elle varie avec l'âge en étant légèrement plus faible chez les enfants les plus âgés [12]. Le même auteur rapporte que près de 50% des femmes en âge de procréer sont anémiques. Ben Salem [10], dans une étude tunisienne, a retrouvé une prédominance féminine avec un sex-ratio de 0,43. L'environnement et le statut socio-économique, avec une carence plus fréquente en cas de niveau socio-économique bas, comme le montre l'étude de l'OMS : en Inde l'anémie est estimée à plus 88% chez les femmes en âge de procréer contre 50% en Afrique et 40% en Amérique latine [8]. La résidence en milieu rural semble légèrement augmenter la prévalence de l'anémie, d'environ 8%. Cet effet du milieu de vie semble varier selon les pays: dans un travail mené au Mali et au Bénin, les auteurs ont mis en évidence un risque d'anémie accru en milieu rural au Bénin, mais diminué au Mali [16]. Dans une étude cas témoin réalisée chez les enfants au Malawi, il a été montré une relation étroite entre l'anémie sévère et la présence d'une bactériémie, le paludisme, une ankylostomiase, infection à VIH, le déficit génétique en G6PD ou encore carence en vitamine A et vitamine B12 [17]. Le Bilan à visée étiologique est fondamental et doit être entrepris systématiquement et l'orientation diagnostique dépendra du sexe et de l'âge. Dans notre série, l'origine digestive prédomine (Tableau 3). Chez la femme en période d'activité gynécologique, lorsque l'interrogatoire retrouve des menstruations certainement pathologiques, l'avis gynécologique est justifié et d'autres investigations, en particulier digestives, sont inutiles. Dans notre étude, toutes les femmes en âge de procréation et les femmes ménopausées ayant des méno-métrorragies ont bénéficié d'une consultation gynécologique. Dans les autres circonstances il faut rechercher un éventuel saignement digestif. Le bilan comportera deux examens pouvant être pratiqués au cours d'une analgésie modérée : Une fibroscopie oeso-gastro-duodénale avec biopsie systématique (++) de la muqueuse duodénale, meilleur moyen de dépister une maladie cœliaque et de la muqueuse gastrique à la recherche d'éventuelle gastrite à Helicobacter pylori. 76/80 de nos patients ayant une anémie ferriprive ont bénéficié d'une FOGD ce qui nous a permis d'étiqueter 26% des AF recensés. Une colonoscopie, en cas de négativité de ce premier bilan, faite chez 13 de nos patients. Quand il existe des troubles digestifs inexpliqués ou une carence martiale récidivante, il faut rechercher une éventuelle hémorragie au niveau de l'intestin grêle et on demandera alors un entéroscanner, ou une entéro-IRM, une entéroscopie voir un examen par vidéo-capsule. Quand aucune étiologie n'est retrouvée, il peut s'agir, chez la patiente en période d'activité génitale, d'une conjonction de facteurs (règles abondantes, grossesses répétées, apport alimentaire insuffisant) ou d'un saignement occulte dont le bilan étiologique reste négatif. Il faut aussi évoquer une pathologie psychiatrique (pathomimie). La surveillance doit être rigoureuse et un nouveau bilan s'impose en cas d'inefficacité du traitement martial ou de récidive et il ne faut pas hésiter à réaliser des nouvelles explorations digestives à distance des premières. Ruivard [9], dans son enquête étiologique après avoir exclu les femmes de moins de 50 ans, a retrouvé 24% de cause digestives basses, 36% liés à une origine digestive haute. Aucune cause n'a été objectivée dans les autres cas lors du premier bilan étiologique. Selon Ben Salem [10], les étiologies sont dominées par les pertes gynécologiques (21.4%), pertes digestives (12.8%) et les carences d'apport (15%). Pour Ben Ahmed [7], la carence martiale est liée le plus souvent à une alimentation carencée dans 83%, une cause gynécologique (8,4%) ou un saignement digestif noté dans 8,4% des cas. Les anémies mégaloblastiques viennent au deuxième rang retrouvées chez 32 patients soit 21,33% de l'ensemble des étiologies avec un déficit en vitamine B12 dominant (31/32). Ben Salem [18], dans une étude portant sur 93 patients admis pour anémie macrocytaire, un déficit en vitamine B12 était confirmé chez 38 patients et en folates dans 6 cas. Pour Ben Ahmed, dans une étude dans la région du Cap Bon tunisien portant sur 40 cas [7], la carence en vitamine B12 était à l'origine de 12,5% des cas d'anémie. Le sex-ratio H/F dans notre série était de 0,5 alors qu'il est de 0.47 pour Ben Salem contre 0,82 pour Ben Ahmed. La moyenne d'âge dans notre groupe est de 50,47 ans contre 56,7 pour Ben Salem. Elle est de 57,7 ± 18 ans pour Ben Ahmed. Dans une étude récente portant sur les patients admis pour anémie mégaloblastique [19], Kechida a retrouvé un âge moyen de 63 ans et un sex-ratio H/F de 1.08. Tous les patients avaient une carence en vitamine B12 secondaire à une maladie de Biermer chez 82% des malades. Les causes non Biermeriennes étaient représentées par une non dissociation de la vitamine B12 de ses protéines porteuses dans 88% des cas et une maladie cœliaque dans 2% des cas. Pour Médaoud [20], le sex-ratio était de 0,91 sur un effectif de 23 patients, l'âge moyen est de 53 ans. Les étiologies sont attribuées à une maladie de Biermer (10) attestées par la présence d'anticorps anti cellules pariétales dans 70%, une gastrite chronique atrophique (9), une carence d'apport (1), une maladie cœliaque (1), une iatrogénie impliquant la colchicine (1) et une pullulation sur diverticulose (1). L'anémie hémolytique est retrouvée au quatrième rang des anémies dans notre série. On a recensé 11 cas, soit 7,33% de nos patients avec sex-ratio H/F de 3/8. Ben Ahmed dans sa série [7], les anémies hémolytiques ne représente que 2,5%. Quatrième étiologie par ordre de fréquence, les SMD ont été recensée chez 9 patients dont la majorité était des hommes (8 hommes). Ben Salem [18] dans son étude incluant 93 patients atteints d'Anémie macrocytaire, un syndrome myélodysplasique a été retrouvé dans 9 cas.

**Tableau 3:** étiologies des anémies ferriprives rencontrées dans notre série

Etiologie	Nombre de cas	Pourcentage(%)
Varices œsophagienne	6	6,6
Ulcère bulbaire	2	2,2
Ulcère gastrique	1	1,1
Œsophagite	3	3,3
Maladie de crohn	2	2,4
Métrorragies fonctionnelles	4	4,4
Fibrome utérin	2	2,2
Néo du col	1	1,1
Gastrite	3	3,3
Maladie cœliaque	4	5,1
Hémorroïdes	8	8,9
Hernie hiatale	1	1,1
Cancer colorectal	1	1,1
Non identifiée	52	57,7

## Conclusion

La survenue d'une anémie chez l'adulte peut représenter un véritable défi diagnostique pour l'interniste et cela parfois dans un contexte d'urgence. La démarche diagnostique doit reposer en priorité sur les données de l'anamnèse, sur l'analyse précise de l'hémogramme et du frottis sanguin. En l'absence de cause évidente, le recours à des examens plus spécialisés s'imposent. Les anémies nutritionnelles par carence martiale et carence en vitamine B12 sont les plus fréquentes et nécessitent l'application immédiate d'une action préventive ciblant en particulier les sujets âgés et socialement démunis.

### Etat des connaissances actuelle sur le sujet

- L'anémie est une pathologie fréquente;
- L'anémie carentielle est le type dominant.

### Contribution de notre étude à la connaissance

- L'anémie ferriprive touche non seulement les pays en développement mais aussi la première étiologie des anémies dans les pays industrialisés;
- Intérêt d'une action préventive ciblant en particulier les sujets âgés, la femme en âge de procréer, l'enfant et les personnes socialement démunies.
